# Metastatic colorectal cancer cells from patients previously treated with chemotherapy are sensitive to T-cell killing mediated by CEA/CD3-bispecific T-cell-engaging BiTE antibody

**DOI:** 10.1038/sj.bjc.6605364

**Published:** 2009-12-01

**Authors:** T Osada, D Hsu, S Hammond, A Hobeika, G Devi, T M Clay, H K Lyerly, M A Morse

**Affiliations:** 1 Department of Surgery, Duke University Medical Center, 401 MSRB, Research Drive, Durham, NC 27710, USA; 2 Program in Molecular Therapeutics, Duke University Medical Center, 401 MSRB, Research Drive, Durham, NC 27710, USA; 3 Duke Comprehensive Cancer Center, Duke University Medical Center, 401 MSRB, Research Drive, Durham, NC 27710, USA; 4 Department of Medicine, Duke University Medical Center, 401 MSRB, Research Drive, Durham, NC 27710, USA; 5 Duke Institute for Genome Sciences & Policy, Duke University Medical Center, 401 MSRB, Research Drive, Durham, NC 27710, USA; 6 MedImmune, LLC, One MedImmune Way, Gaithersburg, MD 20878, USA; 7 Department of Pathology, Duke University Medical Center, 401 MSRB, Research Drive, Durham, NC 27710, USA; 8 Department of Immunology, Duke University Medical Center, 401 MSRB, Research Drive, Durham, NC 27710, USA

**Keywords:** bispecific antibody, BiTE, carcinoembryonic antigen, cytolytic killing

## Abstract

**Background:**

Novel technologies to redirect T-cell killing against cancer cells are emerging. We hypothesised that metastatic human colorectal cancer (CRC) previously treated with conventional chemotherapy would be sensitive to T-cell killing mediated by carcinoembryonic antigen (CEA)/CD3-bispecific T-cell-engaging BiTE antibody (MEDI-565).

**Methods:**

We analysed proliferation and lysis of CEA-positive (CEA+) CRC specimens that had survived previous systemic chemotherapy and biologic therapy to determine whether they could be killed by patient T cells engaged by MEDI-565 *in vitro*.

**Results:**

At low concentrations (0.1–1 ng ml^−1^), MEDI-565+ T cells caused reduced proliferation and enhanced apoptosis of CEA+ human CRC specimens. High levels of soluble CEA did not impair killing by redirected T cells and there was no increase in resistance to T-cell killing despite multiple rounds of exposure.

**Conclusions:**

This study shows for the first time that metastatic CRC specimens derived from patients previously treated with conventional chemotherapy can be lysed by patient T cells. Clinical testing of cancer immunotherapies, such as MEDI-565 that result in exposure of tumours to large numbers of T cells, is warranted.

Antigen-specific cytotoxic T cells (CTLs) have the capacity to kill human cancers, as showed by tumour regression after adoptive transfer of *ex vivo-*expanded tumour-infiltrating lymphocytes (TILs) and T-cell receptor gene-transfected T cells to melanoma patients (Leen *et al*, 2007). However, generating antigen-specific T cells *in vitro* for adoptive transfer is complicated, time-consuming and at best, is only successful in 70% of TIL cultures (Dudley *et al*, 2003). An alternative is to redirect large numbers of T cells through the use of bispecific antibodies, which target CD3 on T cells and a surface antigen on tumour cells, including bispecific single-chain antibodies of the BiTE class (Wolf *et al*, 2005). These recombinant constructs transiently link resting T cells to tumour cells, leading to T-cell activation and serial lysis of tumour cells (Hoffmann *et al*, 2005; Brandl *et al*, 2007; Brischwein *et al*, 2007). Mouse models have shown high levels of activity of BiTE antibodies targeted against EphA2 and CD19 (Dreier *et al*, 2003; Schlereth *et al*, 2006; Brischwein *et al*, 2006; Offner *et al*, 2006; Hammond *et al*, 2007). Witthauer *et al* (2008) reported that a BiTE targeting EpCAM could lead to T-cell-mediated killing of breast cancer cells in pleural effusions. Recently, Bargou *et al* (2008) reported clinical activity and a safety profile suitable for continued development of the BiTE antibody blinatumomab (also called MT103/MEDI-538) with dual specificity for CD19 and CD3 in a study of non-Hodgkin's B-cell lymphoma patients who had experienced relapse after standard therapies.

MEDI-565, also known as MT111, is composed of a human single-chain antibody recognising carcinoembryonic antigen (CEA, CD66e and CEACAM5), which is frequently expressed in carcinomas of the lung, pancreas, stomach, ovary, uterus, breast, colon and rectum (Hammarstrom, 1999), and a de-immunised single-chain antibody specific for CD3, which is connected by a short flexible linker sequence (Lutterbuese *et al*, 2009). Conventional CEA-specific antibodies can bind with high affinity and selectivity to CEA-expressing (CEA+) tumours *in vivo* but they do not recognise CEA expressed on the luminal side of several normal epithelial tissues, thus limiting their potential toxicity (Mayer *et al*, 2000). The bispecific BiTE antibodies, CEA/CD3, were recently shown to prevent subcutaneous tumour growth and formation of lung metastases in preclinical models (Lutterbuese *et al*, 2009).

The purpose of our study was to determine whether T cells from normal donors or cancer patients could be redirected by MEDI-565 to kill human colorectal cancer specimens that had survived previous chemotherapy treatment. We aimed to confirm the function of MEDI-565 with the T cell of cancer patients and against human colorectal cancers. Furthermore, we aimed to confirm that the mechanism of killing by MEDI-565 was similar to that of other forms of T-cell-mediated killing. Finally, we wanted to determine whether tumours remained sensitive to repeated rounds of exposure to MEDI-565. We found that tumour cells, from patients previously treated with chemotherapy, had their growth inhibited and underwent apoptotic cell death when exposed to combinations of T cells with MEDI-565.

## Materials and methods

### Reagents

Fluorescein isothiocyanate-anti-lineage cocktail 1 mAb, PerCP-anti-CD4 mAb, APC-anti-CD8 mAb, PE-anti-CD69 mAb, PE-anti-CD25 mAb, FITC-anti-granzyme B mAb, PE-anti-Fas ligand mAb and streptavidin-APC were purchased from BD Biosciences (San Jose, CA, USA). Fluorescein isothiocyanate-labelled and PE-labelled anti-CEA mAbs were from Sanquin (Amsterdam, The Netherlands), and 7-AAD and Annexin V-biotin kit were purchased from Immunotech (Marseille, France, cat no. PN IM3422). Ethylene glycol tetraacetic acid (EGTA) was obtained from Sigma (St Louis, MO, USA), and purified human CEA protein was purchased from TriChem (West Chester, PA, USA).

### Construction and production of bispecific antibodies

Standard DNA technologies were used to construct MEDI-565, also known as MT111, and the control MEC14 BiTE, as described (Lutterbuese *et al*, 2009). The selection of MEDI-565 was from a set of bispecific single-chain antibodies (Lutterbuese *et al*, 2009) and possessed a set of characteristics identified as important for development of a biological drug. It is composed of a humanised anti-CEA single-chain antibody (Chester *et al*, 1994) and a ‘de-immunised’ human CD3*ε*-specific single-chain antibody derived from the mouse monoclonal antibody L2K (Brischwein *et al*, 2006). MEC14 BiTE is composed of a murine anti-Mecoprop (an herbicide) single-chain antibody linked to the same anti-CD3*ε* single-chain antibody used to construct MEDI-565. The expression vector pEF-DHFR containing the coding sequences of MEDI-565 or MEC14 BiTE (Cont BiTE) was transfected into DHFR-deficient CHO cells. Each antibody was purified from CHO cell culture supernatants using immunobilised metal affinity chromatography and gel filtration essentially as described (Kufer *et al*, 2001). Antibody preparations containing primarily the monomeric form (>97%) of each bispecific antibody was used in all experiments.

### Tumour cell lines

Ls174T (ATCC CCL-188), a CEA highly expressing colon carcinoma cell line, AsPC-1 (ATCC CRL-1682), a CEA+ pancreatic adenocarcinoma cell line and SW480 (ATCC CCL-228) and HCT116 (ATCC CCL-247), the CEA-negative colorectal carcinoma cell lines, were purchased from ATCC (Manassas, VA, USA). The cell lines were cultured in RPMI1640 medium supplemented with 10% fetal bovine serum (Invitrogen Life Technologies, Carlsbad, CA, USA).

### Mice

NOD.CB17-*Prkdcscid*/J (NOD/SCID) mice were purchased from Jackson Laboratory (Bar Harbor, ME, USA) and bred in the Duke Comprehensive Cancer Center Isolation Facility. All work was performed in accordance with the approved protocol of the Duke IACUC.

### Tumour cell isolation from patients’ colorectal cancer specimens and establishment of explants in NOD/SCID mice

Patients undergoing resection of colorectal cancer metastatic to the liver, which were refractory to standard chemotherapy (including fluorouracil, oxaliplatin and bevacizumab), provided signed informed consent approved by the Duke University Medical Center Institutional Review Board before surgery. After the collection of the colorectal cancer specimen, the tissue was minced with a blade into pieces smaller than 2 mm cube and digested overnight with triple enzyme buffer containing collagenase IV (1 mg ml^−1^, Sigma, no. C-5138), hyaluronidase (100 *μ*g ml^−1^, Sigma, no. H-6254) and deoxyribonuclease (20 U ml^−1^, Sigma, no. D-5025) in RPMI1640 medium. The cells were spun down, washed with PBS thrice, resuspended in Hank's balanced salt solution and mixed with Matrigel (BD Biosciences) in a 1 : 1 ratio. Cells (half of available cells from digestion procedure, typically 1 × 10^6^ cells) were injected into the back of NOD/SCID mice. After 2- to 4-month growth *in vivo*, when tumours reached approximately 1 cm in diameter, the mice were killed and the tumours were excised, minced and put into *in vitro* culture. Some of the minced cells were injected into the flank of NOD/SCID mice, and serial *in vivo* passages were performed. Colorectal cancer (CRC) cells growing *in vitro* were used as target cells of the assays. These cells (CRC007, CRC010 and CRC039) were analysed for their HLA class I expression and CEA expression, and were proven to be positive for both molecules.

### Flow-based cytotoxicity assay

T cells were negatively isolated from the PBMCs of the normal donors or patients using a T-cell isolation kit (Invitrogen Dynal AS, Oslo, Norway, cat no. 113.11D). In all experiments, purity of CD3+ cells exceeded 95% of the CD45+ leukocyte population after isolation procedures. For the cytotoxicity assays, 1 × 10^5^ tumour cells and 5 × 10^5^ negatively isolated T cells were put into 96-well U-bottom plates with MEDI-565 or Cont BiTE at concentrations ranging from 0.01 to 10 000 ng ml^−1^. Alternatively, in some experiments using 12-well plates, 5 × 10^5^ tumour cells and 2.5 × 10^6^ T cells were added to each well with MEDI-565 or Cont BiTE. After 1–7 days of incubation, all cells were harvested with 0.05% trypsin/EDTA and spun down by centrifugation. Cells were then stained with anti-CEA-FITC and 7-AAD or propidium iodide, and CEA+ cells were analysed for their viability after acquisition using a FACSCalibur flow cytometer (BD Biosciences). Alternatively, cells were labelled with biotin-conjugated Annexin V, and then stained with anti-CEA-PE, 7-AAD and Streptavidin-APC. The CEA+ cells were analysed for expression of Annexin V as a marker of apoptosis. To test whether cytotoxicity was dependent on exocytosis of cytotoxic granules, the assay was performed in the presence of 4 mM EGTA, a chelator of extracellular calcium required for exocytosis (Lowin *et al*, 1996; Voskoboinik *et al*, 2005, 2006).

### MTT assay

The tumour cells, AsPC-1, were cultured with T cells in a 1 : 5 ratio for 7 days in the presence of MEDI-565 or Cont BiTE (100 ng ml^−1^). On day 7, floating cells were discarded and only adherent AsPC-1 cells were harvested with 0.05% trypsin/EDTA and washed with PBS thrice. 1 × 10^4^ AsPC-1 tumour cells were added to each well of 96-well flat-bottom plates in 200 *μ*l of complete RPMI1640 medium. The cells were allowed to adhere to the plates overnight at 37 °C (day 0) and were further incubated for 1, 2, 4 or 7 days. A total of 20 *μ*l of 10 × 3-(4,5-dimethylthiazol-2-yl)-2,5-diphenyl-tetrazolium bromide (MTT, 5 mg ml^−1^) solution was added to each well, and incubated at 37 °C for 2 h. The adherent cells were lysed with 150 *μ*l of dimethylsulphoxide (DMSO) and the optical density (OD) at 550 nm was measured.

Sensitivity of colorectal cancer cells to oxaliplatin was also determined using MTT assay. In brief, 1 × 10^4^ CRC cells were added to each well of 96-well flat-bottom plates and, after overnight incubation, various concentrations of oxaliplatin were added to the wells. The MTT assay was performed after 72-h incubation with oxaliplatin and the IC_50_ was reported as the concentration at which 50% reduction in cell proliferation occurred.

### Trypan blue dye exclusion assay

To assess MEDI-565 effects on tumour cell proliferation, tumour cells (0.5 × 10^6^ per well) were cultured with or without T cells (2.5 × 10^6^ per well) for 5 or 7 days in 12-well plates in the presence of MEDI-565 or Cont BiTE (each at 100 ng ml^−1^). Photographs were taken at the magnifications of × 40, × 100 and × 200, and the numbers of tumour cell/T-cell clusters, tumour cell attached by at least three lymphocytes, were counted in three random high-power fields ( × 100). Cells were harvested with 0.05% trypsin/EDTA and Trypan blue dye was added after which the sum of viable tumour cells (Trypan blue negative) and dead cells (Trypan blue staining) was determined. Smaller cells (morphologically T cells) were eliminated from counting.

### Cell-cycle analysis

The tumour cells, AsPC-1, were cultured with normal donor's T cells in a 1 : 5 ratio for 7 days in the presence of MEDI-565 or Cont BiTE (100 ng ml^−1^). On day 7, floating cells were discarded and only adherent AsPC-1 cells were harvested from flasks with 0.05% trypsin/EDTA, washed with PBS and resuspended in 0.5 ml of 0.1% glucose/PBS, and cells were then fixed by gently adding 5 ml of cold 70% ethanol. Cells were kept at 4 °C overnight, and then washed with PBS twice. Propidium iodide was added to the cell suspension, and incubated at 37 °C for 30 min. Cells were acquired using FACSCalibur flow cytometer (BD Biosciences), and DNA content was analysed for tumour cells. Tumour cells were gated based on the size and granularity in forward scatter and side scatter histogram.

### Intracellular cytokine staining

2.5 × 10^6^ T cells and 5 × 10^5^ AsPC-1 cells were incubated in the presence of MEDI-565 or Cont BiTE (each at 100 ng ml^−1^) for 1, 2 or 4 days as described above. For the last 5 h of the incubation period, Brefeldin A (1 mg ml^−1^) was added to the medium. Then, EDTA (final concentration, 2 mM) was added to the medium to dissociate cells and after 10 min the cells were harvested, washed with PBS twice and fixed with 1% formaldehyde in PBS. Cells were permeabilised with permeabilising solution (BD Biosciences) for 20 min, and stained with anti-CD4-PerCP, anti-CD8-APC and FITC-labelled anti-granzyme B or PE-labelled anti-Fas ligand. Forward Scatter^low^ Side Scatter^low^ CD4+ and CD8+ T cells were analysed for their granzyme B or Fas ligand expression (Supplementary Figures).

### Enzyme-linked immunosorbent assay

2.5 × 10^6^ T cells and 5 × 10^5^ AsPC-1 cells were incubated in the presence of MEDI-565 or Cont BiTE (each at 100 ng ml^−1^) for 1, 3 or 5 days and supernatants were harvested and kept frozen at −80 °C until used. Supernatants were analysed using ELISA for the presence of granzyme B and perforin (Mabtech Inc., Cincinnati, OH, USA) according to the manufacturer's instructions. Colour was developed with 3,3′,5,5′,tetramethylbenzidine (TMB) stabilised substrate (Promega, Madison, WI, USA), stopped by adding 1 N H_2_SO_4_, and OD at 450 nm was analysed using ELISA plate reader (Bio-Rad, Model 680, Hercules, CA, USA; Supplementary data).

### Th1/Th2 CBA analysis

Culture supernatants were collected after 5 days of T-cell/tumour cell co-incubation with MEDI-565 or Cont BiTE (each at 100 ng ml^−1^). The concentrations of IL-2, IL-4, IL-5, IL-10, TNF-*α* and IFN-*γ* were measured with a BD Cytometric Bead Array Th1/Th2 cytokine kit (BD Biosciences), according to the manufacturer's instructions, and analysed on a FACSCalibur flow cytometer using BD CBA software (BD Biosciences; Supplementary data).

### Statistical analysis

The Student's *t-*test was used to analyse differences in tumour cell killing between MEDI-565 and control MEC14 BiTE-treated tumours. Differences at *P*<0.05 were considered statistically significant.

## Results

### Human CEA+ tumour cells are recognised and undergo apoptosis by normal human donor T cells redirected by MEDI-565

**Figure 1:**
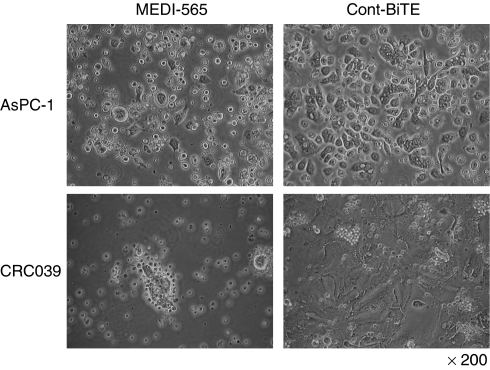
MEDI-565 and T-cell (MEDI-565/T-cell)-mediated morphologic changes of CEA+ cancer cells. Carcinoembryonic antigen-positive human pancreatic cancer cell line, AsPC-1, and a CEA+ human colorectal cancer explant (CRC039) were used as target cells. T cells, negatively isolated from human PBMCs, were used as effector cells. 5 × 10^5^ target cells and 2.5 × 10^6^ T cells were co-cultured in each well of 12-well plates with 100 ng ml^−1^ of MEDI-565 or MEC14 control BiTE (Cont BiTE). Cells were incubated for 5 days at 37 °C, and photographed at × 200 magnification.

To examine the cytolytic effects of MEDI-565, the CEA+ human pancreatic cancer cell line AsPC-1 was incubated with purified, human T cells and MEDI-565 or a control BiTE (which binds the herbicide mecoprop and human CD3; Cont BiTE) for 5 days. As shown in Figure 1, in the presence of MEDI-565, but not Cont BiTE, tumour cells were surrounded by T cells, detached from the bottom of the wells, and showed apoptotic changes in cell morphology. Similar observations were made when using human CEA+ tumours (e.g., CRC039) obtained from resection specimens as target cells. We found a markedly greater number of tumour cells with adherent T cells in the MEDI-565 containing co-cultures compared with the Cont BiTE containing cultures (AsPC-1: 23.0±3.6 *vs* 1.3±0.6 clusters per field, CRC039: 15.7±2.1 *vs* 1.7±1.5 clusters per field, *P*<0.005). These data show that MEDI-565 redirects large numbers of T cells to engage tumour cells.

### Proliferation of human CEA+ tumour cells is inhibited and cytotoxicity is enhanced by T cells redirected by MEDI-565

**Figure 2:**
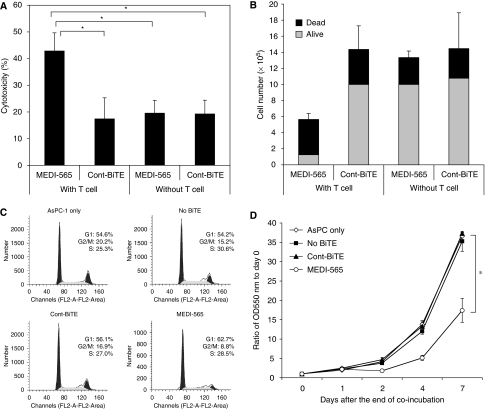
MEDI-565/T-cell inhibits proliferation of CEA+ cancer cells. (**A**) AsPC-1 cells (5 × 10^5^ per well) were cultured with or without T cells (2.5 × 10^6^ per well) for 7 days in 12-well plates in the presence of MEDI-565 or Cont BiTE (100 ng ml^−1^). MEDI-565-induced T-cell cytotoxicity was assessed by staining tumour cells with PE-labelled anti-CEA mAb and 7-AAD. Carcinoembryonic antigen-positive tumour cells were analysed for their 7-AAD positivity. The assay was repeated four times and the average values of cytotoxicity for each condition are shown. ^*^P<0.001 (Student's *t*-test). (**B**) MEDI-565 effect on tumour cell proliferation was assessed by counting cells with Trypan blue dye exclusion method. The assay was performed with duplicated wells for each condition and the sum of viable cells (grey) and dead cells (black) are shown. (**C, D**) AsPC-1 cells (6 × 10^6^ per flask) were incubated with T cells (30 × 10^6^ per flask) for 7 days in 150 cm^2^ flask. Floating cells were discarded and adherent cells were harvested with 0.05% trypsin/EDTA. Cells were washed with PBS, resuspended and then fixed with cold 70% ethanol. Cells were stained with propidium iodide and samples were acquired using a FACSCalibur flow cytometer and DNA content of tumour cells were analysed. The percentages of G0/G1, G2/M and S phases are shown in each histogram (**C**). Harvested AsPC-1 cells (1 × 10^4^ per well) were put into 96-well flat-bottom plates on day −1, and incubated overnight. On days 0, 1, 2, 4 and 7, the 3-(4,5-dimethylthiazol-2-yl)-2,5-diphenyl-tetrazolium bromide (MTT) assay was performed. In brief, 20 *μ*l of 10 × MTT (5 mg ml^−1^) solution was added to each well, and incubated at 37 °C for 2 h. Adherent cells were lysed with 150 *μ*l of dimethylsulphoxide (DMSO) at 37 °C for 5 min, and OD 550 nm was measured using an ELISA plate reader. Value of day 0 was used as a baseline data, and the ratios of OD550 nm values of each time point were plotted (**D**). ^*^P<0.001 (Student's *t-*test).

We analysed the cytotoxicity of tumour cells caused by MEDI-565 using flow cytometric analysis to detect uptake of a dye into killed tumour cells. As shown in Figure 2A, the cytotoxicity of MEDI-565 required the presence of T cells, suggesting that MEDI-565 alone does not cause cell death. We next analysed whether MEDI-565 reduced proliferation of tumour cells in the presence of T cells by counting viable cells with Trypan blue dye exclusion. As shown in Figure 2B, MEDI-565 significantly inhibited the cell proliferation and resulted in only one-eighth of the number of viable tumour cells in culture after a 7-day incubation period (*P*<0.05). Interestingly, T cells proliferated in MEDI-565 containing co-cultures, but not in Cont BiTE containing co-culture (3.31±0.49 × 10^6^ *vs* 0.72±0.11 × 10^6^ cells per well), possibly because of activation as evidenced by upregulation of CD69 and CD25 observed only in MEDI-565 cultures. The expression (percent positivity/mean fluorescence intensity (MFI)) of CD69 and CD25 by T cells in a representative MEDI-565 culture was 48.3%/301.5 and 20.3%/222.8, respectively, whereas it was 1.5%/10.6 and 9.3%/36.4, respectively, in Cont BiTE cultures.

To evaluate the effect of MEDI-565 on the cell cycle of tumour cells, we stained tumours with propidium iodide after 7 days of incubation in the presence of T cells with or without MEDI-565 or Cont BiTE (Figure 2C). Only tumour cells cultured with MEDI-565+ T cells had substantially smaller number of cells in G2/M phase compared with all other conditions tested. The proliferation of these cells for 7 days after treatment was also monitored using MTT assay. As shown in Figure 2D, AsPC-1 tumour cells previously exposed to MEDI-565+ T cells proliferated significantly slower than tumour cells from other conditions, suggesting an anti-proliferative effect of MEDI-565.

### Normal donor- and cancer patient-derived T cells with MEDI-565 induce comparable cytotoxicity against CEA+ cancer cell lines in a CEA-specific manner

**Figure 3:**
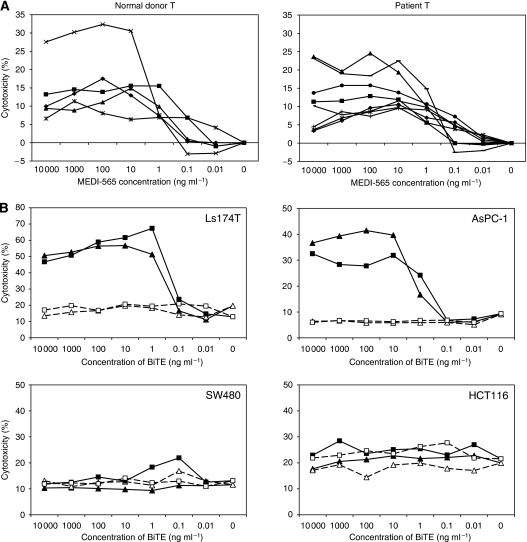

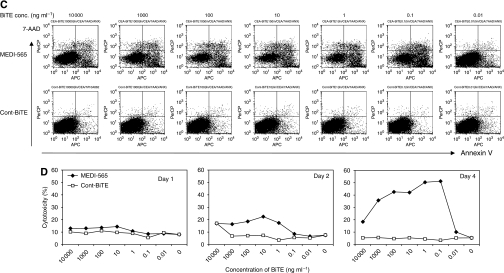
MEDI-565/T-cell-induced apoptotic cell death of a CEA+ tumour cell line. (**A**) MEDI-565 induced cytotoxic capacity of T cells derived from colorectal cancer patients compared with normal healthy donor T cells. T cells were isolated from the PBMCs derived from normal healthy donors (left panel) or colorectal cancer patients (right panel) using T-cell-negative isolation kit. Isolated T cells were co-cultured with AsPC-1 tumour cells in a 5 : 1 effector-to-target ratio in the presence of various concentrations of MEDI-565 (0.01∼10 000 ng ml^−1^) in 96-well U-bottom plates as described in Materials and Methods. Cells were incubated for 2 days, and the cytotoxicity was analysed using flow cytometry with 7-AAD staining. No significant difference (*P*>0.05 for Student's *t*-test, see text) in MEDI-565-induced T-cell cytotoxicity was observed between T cells from cancer patients and T cells from normal donors when compared at each concentration of MEDI-565. (**B**) Carcinoembryonic antigen (CEA) specificity of MEDI-565/T-cell mediated killing of tumour cells. The CEA+ tumour cell lines (Ls174T and AsPC-1) and CEA-negative tumour cell lines (SW480 and HCT116) were incubated in a 96-well U-bottom plate with T cells negatively isolated from the PBMCs of normal donors (triangle symbols) or cancer patients (square symbols). MEDI-565 or Cont BiTE was put into the culture at the indicated concentrations. After 2 days of incubation, cells were harvested from the plates with 0.05% trypsin/EDTA and stained with FITC-labelled anti-lineage marker and 7-AAD. The 7-AAD-positive cells in lineage marker negative cells are analysed and shown in each line graph. (**C, D**) Effects of time and concentration of MEDI-565 on T-cell-induced apoptosis of CEA+ tumour cells. Both the AsPC-1 cells (1 × 10^5^ cells per well) and normal donor's T cells (5 × 10^5^ cells per well) were put into a 96-well U-bottom plate with indicated concentrations of MEDI-565 or Cont BiTE. After 4 days of incubation, cells were harvested with 0.05% trypsin/EDTA and labelled with biotin-labelled Annexin V, and then stained with FITC-anti-lineage mixture, PE-anti-CEA, 7-AAD and streptavidin-APC. Lineage marker-negative and CEA+ tumour cells were analysed for their 7-AAD/Annexin V staining. (**C**) The AsPC-1 cells were incubated with T cells in the presence of MEDI-565 or Cont BiTE (100 ng ml^−1^) for 1, 2 or 4 days and stained as described in (**B**). Percentages of annexin V-positive cells (including 7-AAD negative and 7-AAD positive) in CEA+ tumour cells are shown in each line graph.

Our previous experiments used T cells from healthy human donors as effector cells for the MEDI-565 antibody. In this study we tested whether circulating T cells from colorectal cancer patients would similarly function with MEDI-565 in lysing CEA+ cancer cells. T cells from five normal donors and eight colorectal cancer patients were used as effector cells and incubated with AsPC-1 cells in a 5 : 1 E : T ratio. Cells were incubated for 2 days, and the cytotoxicity was analysed using flow cytometry with 7-AAD staining. As shown in Figure 3A, T cells from cancer patients (right panel) caused comparable cytotoxicity as T cells from healthy volunteer donors (left panel) in the presence of the MEDI-565 antibody. There were no statistically significant differences in induced cytotoxicities between these two T-cell sources when compared at each MEDI-565 concentration (*P*=0.73, 0.35, 0.45, 0.52, 0.83, 0.79 and 0.58 for 10^4^, 10^3^, 10^2^, 10^1^, 10^0^, 10^−1^ and 10^−2^ ng ml^−1^ BiTE concentration, respectively, using Student's *t-*test). These data suggest that cancer patient T cells do not have an intrinsic deficit in their killing potential when engaging tumours through MEDI-565.

To confirm that cytotoxicity was CEA specific, we tested both CEA+ (Ls174T and AsPC-1) and CEA− (SW480 and HCT116) cell lines in the presence of T cells and MEDI-565 or Cont BiTE (Figure 3B). MEDI-565-induced T-cell cytotoxicity was only observed with CEA+ cell lines, indicating surface antigen specificity mediated by MEDI-565. In addition, cancer patient-derived T cells showed comparable cytotoxicity against CEA+ cancer cell lines, supporting the potential use of MEDI-565 in patients with CEA+ cancers.

### Normal donor- and cancer patient-derived T cells with MEDI-565 induce apoptotic cell death of a CEA+ cancer cell line

To examine the effect of MEDI-565 and T cells on apoptotic cell death of tumour cells, AsPC-1 cells were incubated with T cells and a range of MEDI-565 concentrations. On study days 1 to 4, subpopulations of tumour cells undergoing apoptosis were identified by flow cytometry using annexin V and 7-AAD (Figure 3C). Figure 3C shows the analysis at day 4 with predominantly early apoptosis (annexin V+ and 7-AAD−) with some component of late apoptosis/cell death (annexin V+ and 7-AAD+) over a wide range of MEDI-565 concentrations (0.1–1000 ng ml^−1^). Apoptosis was not observed until day 2, but reached a maximum at day 4 (Figure 3D) and did not consistently increase further by day 7 (data not shown).

### CEA+ human metastatic colorectal tumour explants are recognised and undergo reduced proliferation and apoptotic cell death when exposed to T cells redirected by MEDI-565

**Figure 4:**
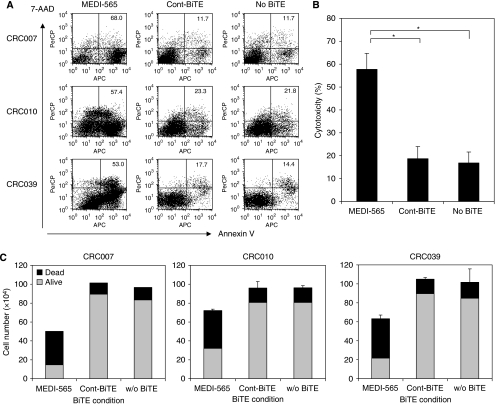
MEDI-565/T-cell-mediated apoptosis of CEA+ human metastatic colorectal cancer explants. (**A**) Colorectal cancer cells from liver metastatic lesions, expanded NOD/SCID mice, excised and maintained in culture as described in the Materials and Methods, were incubated with MEDI-565 or MEC14 control BiTE (100 ng ml^−1^). After 5 days of incubation, cells were harvested with 0.05% trypsin/EDTA, washed and stained with biotin-annexin V and streptavidin-APC, FITC-anti-lineage marker, PE-anti-CEA and 7-AAD. Annexin V/7-AAD positivity was evaluated in lineage marker-negative, CEA+ tumour cell populations using flow cytometry. The percentages of annexin V-positive cells are shown in each dot plot. (**B**) Co-incubation of T cells and colorectal cancer cells (CRC007, CRC010 and CRC039) together with MEDI-565 or Cont BiTE was repeated twice, and the average percentages of apoptotic tumour cells (Annexin V+) are shown in the bar graph. ^*^P<0.005 (Student's *t-*test). (**C**) Cells were counted with Trypan blue dye exclusion method. The numbers of viable cells (grey) and dead cells (black) are shown in each bar graph. The error bars show s.d. of the total number of cells calculated from duplicate wells.

To provide a more clinically relevant assessment of MEDI-565 activity, we used the short-term cultured human metastatic colorectal cancer cells derived from surgical resections of patients previously treated with oxaliplatin and fluorouracil-containing regimens. The colorectal cancer cells had been implanted into NOD/SCID mice, grown, removed and then maintained *in vitro*. Flow cytometric and immunohistochemical analyses showed that these metastatic tumour explants expressed CEA, and had histological characteristics of metastatic colorectal cancers (data not shown). In addition, these explants (CRC007, CRC010 and CRC039) were tested for their sensitivity to oxaliplatin *in vitro* and were found to have IC_50_ of 2.5, 12.0 and 30.5 *μ*M, respectively. Compared with the IC_50_ in a screen of colorectal cancer cell lines treated with oxaliplatin (LS180, <0.3 *μ*M; Colo205, <0.3 *μ*M; and HCT8, <0.3 *μ*M), these explants were determined to be relatively insensitive to oxaliplatin. These metastatic tumour explants were incubated with T cells isolated from healthy volunteer donors for 5 days (Figure 4 and Supplementary Figure 1). Metastatic tumour explants cultured with MEDI-565, but not Cont BiTE, were surrounded by T cells, detached from the bottom of the well, and were gradually killed (Supplementary Figure 1). We confirmed apoptotic cell death of cells from the metastatic tumour explants incubated with MEDI-565 and T cells by annexin V and 7-AAD staining using flow cytometry at day 5 (Figures 4A and B). The average values of cytotoxicity of these three colorectal cancer explants (CRC007, CRC010 and CRC039) from repeated assays are shown in a bar graph (Figure 4B). MEDI-565 induced significantly higher apoptotic cell death of CEA+ colorectal tumour explants when compared with Cont BiTE or without BiTE antibody condition (*P*<0.005). These data also indicate that, despite being insensitive to oxaliplatin, human colorectal cancer cells are sensitive to killing mediated by MEDI-565.

To assess the combined effects of reduced cell growth and induction of apoptotic cell death, we also analysed the number of live and dead cells using Trypan blue dye exclusion. Similar reductions in total cells for all three cancer cell cultures were observed (Figure 4C). Viable cancer cells in the presence of MEDI-565 and T cells were reduced by 66–80% relative to Cont BiTE/T cells, or no treatment. Thus, treatment of cultures with MEDI-565 combined with T cells induced both anti-proliferative and/or apoptotic effects on human metastatic tumour cells, resulting in a massive reduction in the total number of viable tumour cells after treatment compared with untreated tumour cells.

### Granzyme B and FasL have a role in MEDI-565/T-cell-induced apoptosis

It is known that T cells kill tumours by the perforin/granzyme B and the Fas/FasL pathways. Therefore, we aimed to confirm that these pathways were also potentially involved in MEDI-565-induced apoptosis. Indeed, we observed greater percentages of granzyme B-and FasL-expressing CD8+ T cells after co-culture of tumours, T cells and MEDI-565 (Supplementary Figures 2 and 3) when compared with control-BiTE. Interestingly, the MEDI-565 induced gradual secretion of perforin by T cells, which corresponded to the slow induction of apoptosis in target cells. These results indicate that the perforin/granzyme B system has an important role in MEDI-565-induced T-cell cytotoxicity.

We next evaluated the cytokines secreted by T cells responding to and destroying the CEA+ tumour cells in cultures containing MEDI-565. Using beads to capture the cytokines, we showed that IL-2, IL-4, IL-5, IL-10, TNF-*α* and IFN-*γ* were all secreted, but IFN-*γ* was found in the highest amount, suggesting that the T cells do become activated and secrete cytokines when activated by MEDI-565 bound to CEA on tumour cells; both Tc1 (Th1) and Tc2 (Th2) type cytokines were detected (Supplementary Figure 4).

### Soluble CEA protein does not affect apoptosis induced by T cells redirected by MEDI-565

**Figure 5:**
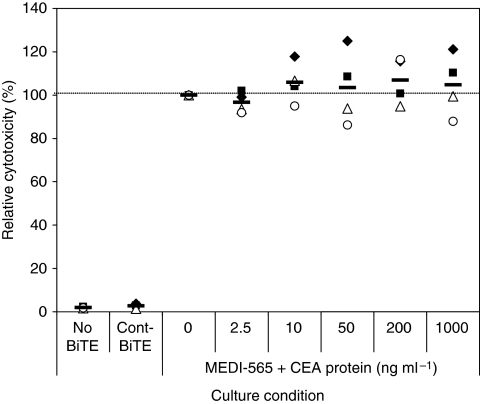
Soluble CEA protein does not affect MEDI-565/T-cell-mediated apoptosis of CEA+ cell line and colorectal cancer cells. AsPC-1 cells or CRC010 colorectal cancer cells (5 × 10^5^ cells per well) were incubated with T cells (2.5 × 10^6^ cells per well) in a 12-well plate in the presence of MEDI-565 (100 ng ml^−1^; 1.8 nM) with the indicated concentration of soluble CEA antigen in the medium (2.5∼1000 ng ml^−1^; 0.01 to 5 nM). After 5 days of incubation, tumour cells were harvested and stained as described in the Figure 4 legend. Annexin V-positive cells in lineage marker-negative, CEA+ tumour cells were evaluated. The cytotoxicity value of the culture with MEDI-565 without soluble CEA protein was set as 100%, and each cytotoxicity value is shown as a percentage relative to this MEDI-565-positive, CEA protein-negative culture. The assay was repeated twice for both AsPC-1 cells and CRC010 cancer cells (filled symbols indicate AsPC-1 cells and open symbols indicate CRC010 tumour cells; each symbol represents an individual T-cell donor). The bars show the average cytotoxicities of each condition.

As many colorectal cancer patients have large amounts of soluble circulating CEA protein in their serum, the MEDI-565 antibody might be competitively inhibited by binding soluble CEA before it can bind membrane-bound CEA on tumour cells. We analysed whether soluble CEA protein in clinically relevant concentrations could affect MEDI-565-induced T-cell cytotoxicity in our assay system. To the cell culture, CEA protein was added at concentrations from 2.5 to 1000 ng ml^−1^, and AsPC-1 cells and CRC010 colorectal cancer cell were used as target cells. Apoptosis at each condition was compared with the apoptosis in MEDI-565/T-cell culture without CEA protein (0 ng ml^−1^) and shown as a percentage (Figure 5). In the tested range of soluble CEA protein, there was no significant competitive inhibition of MEDI-565-induced cytotoxicity. This result indicates that MEDI-565 may not be competitively inhibited by elevated soluble CEA levels in the serum of cancer patients.

### CEA+ cancer cell lines remain sensitive to T-cell-mediated killing despite repeated exposure to T cells and MEDI-565

**Figure 6:**
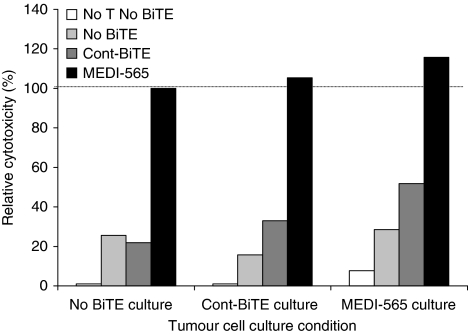
Repeated treatment with MEDI-565 and T cells does not abolish tumour cell susceptibility to MEDI-565/T-ell-mediated apoptosis. AsPC-1 tumour cells (6 × 10^6^) were cultured with T cells (3 × 10^7^) in 150 cm^2^ T flasks with MEDI-565 or Cont BiTE (100 ng ml^−1^). Additional BiTE molecules were added every 3 days, and after 10-day incubation, floating cells were washed out and cells were allowed to expand for 7 days (one cycle). Three cycles of attack/rest were repeated for each condition over 50 days. Adherent AsPC-1 cells were harvested and used as target cells for cytotoxicity assay. After 5 days of incubation with T cells, tumour cells were stained as described in the Figure 4 legend. Annexin V-positive cells in lineage marker-negative, CEA+ tumour cells were evaluated. The cytotoxicity value of the incubation with AsPC-1 target cells from long-term culture without BiTE molecule was set as 100%, and each cytotoxicity value is shown as a percentage relative to this condition. The assay was repeated twice with similar results.

Many anticancer drugs will induce outgrowth of drug-resistant cancer cells, and repeated treatments may become ineffective. To test whether we could repeatedly treat tumour cells with MEDI-565 and T cells, we created an analogous *in vitro* model in which AsPC-1 cells were exposed to three cycles of killing by T cells in the presence of MEDI-565 or Cont BiTE. Harvested cells were analysed using flow cytometry for their susceptibility to MEDI-565/T-cell-mediated apoptosis and levels of CEA expression each cycle. As shown in Figure 6, AsPC-1 cells repeatedly exposed to MEDI-565/T cell maintained their susceptibility to MEDI-565/T-cell-mediated apoptosis at a similar level to non-treated AsPC-1 cells. It should be noted that the CEA expression levels on the AsPC-1 cells remained constant throughout the assay period (data not shown). On the basis of this result, MEDI-565/T cells may not induce escape mechanisms in cancer cells, and thus might be suitable for long-term and repeated treatment.

## Discussion

The purpose of this study was to determine whether CEA+ metastatic colorectal cancers from patients previously treated with chemotherapy, but in whom the tumour was not eradicated, could be recognised and killed by T cells. Using a novel technology to redirect the specificity of T cells, we evaluated the efficacy of a CEA/CD3- bispecific antibody of the BiTE class against human colorectal cancer cell lines and metastatic colorectal cancer explants. We confirmed that T-cell-mediated tumour cell death occurred at very low concentrations of MEDI-565 (1 ng ml^−1^) and without the addition of costimulatory agents. Similar levels of MEDI-565-redirected T-cell lysis were observed when T cells from colorectal cancer patients or from normal healthy donors were used. Importantly, MEDI-565/T-cell treatment could efficiently induce cell death of colorectal cancer cells derived from different patients. We found that anti-proliferative activity required MEDI-565, T cells and target cells expressing CEA. T cells alone or T cells with Control BiTE did not show these effects on CEA-expressing cancer cells.

The results of our study are noteworthy for several reasons. First, we showed that metastatic colorectal cancers, which had survived previous conventional systemic therapy, could be killed by a T-cell-mediated therapy. In addition, we analysed the sensitivity of these tumour cells to oxaliplatin *in vitro* and found that the IC_50_ (2.5 12.0 and 30.5 *μ*M) for inhibition of proliferation was higher than the majority of 25 colorectal cancer cell lines we had previously tested for sensitivity to oxaliplatin (such as LS180, Colo205 and HCT8, which are considered sensitive with IC_50_ <0.3 *μ*M). Therefore, we believe that the colorectal cancer explants are relatively oxaliplatin-insensitive tumour cells.

Second, the low concentrations of MEDI-565 required for activity suggest a potent anti-tumour capacity (Figure 3). This low concentration has been reported for other BiTE antibodies. Bargou *et al* (2008) showed the safety of a BiTE antibody, blinatumomab (a BiTE with dual specificity for CD19 and CD3), in their clinical trial, and confirmed that responses to blinatumomab as a single agent occurred in B-cell lymphoma patients at a serum level of 0.6 ng ml^−1^. This is about five orders of magnitude below serum levels reported for the monoclonal antibody rituximab at standard doses, which similarly elicits objective responses as a single agent in this disease (Hainsworth *et al*, 2003; Ghielmini *et al*, 2005). The enormous potency difference between the BiTE blinatumomab and a conventional antibody could be explained by a greater number of T cells attacking an individual tumour cell (both due to more T cells that are able to recognise tumour and proliferation of the engaged T cells) or more efficient lysis of tumour cells by the engaged T cells (due to activation of T-cell-mediated killing by engagement of only very few CD3 receptor subunits). Interestingly, we observed the activation of both CD4 and CD8 T cells against tumour cells in the presence of MEDI-565 (Supplementary Figures 2 and 3). Consistent with our findings, Kischel *et al* (2009) showed that both CD4 and CD8 T cell subset can contribute to redirected target cell killing with EpCAM/CD3-BiTE antibody. Thus, the high potency of BiTE molecule may derive from a broader spectrum and a greater number of effector T cells engaged to kill target cells. We also observed that the number of T cells in the MEDI-565 co-cultures was greater than the number placed into the cultures and was also greater than that in the control BiTE cultures after 5 days, suggesting that there was proliferation of the effector T cells. Finally, there was upregulation of CD69 on these T cells, suggesting that they were activated.

Third, we showed that resistance to T-cell-mediated killing may not occur. We created an *in vitro* model of repeated MEDI-565/T cell exposure, by repeating three cycles of MEDI-565/T-cell attack and rest with the CEA-expressing cell line AsPC-1. We observed that these cells maintained their susceptibility to MEDI-565/T-cell attack after prolonged treatment *in vitro* (Figure 6). Interestingly, CEA expression of tumour cells was retained despite prolonged treatment, suggesting that MEDI-565/T-cell treatment may not easily induce escape mechanisms in cancer cells. Hypothetically, re-treatment with MEDI-565 can be still effective for progressive disease that was once treated with MEDI-565 successfully at its earlier stages.

Fourth, we were concerned that the high circulating levels of CEA could impair MEDI-565 function through competitive inhibition of binding to surface CEA. As many colorectal cancer patients have high levels of soluble CEA protein in their serum (Goldstein and Mitchell, 2005), an antibody may be less effective in those patients. We studied whether soluble CEA inhibited the function of MEDI-565. At clinically relevant concentrations of soluble CEA (2.5–1000 ng ml^−1^), 100 ng ml^−1^ of MEDI-565 induced similar levels of T-cell-mediated cell death compared with vehicle control, suggesting no major inhibitory effect of soluble CEA on MEDI-565 function (Figure 5). MEDI-565 does bind to soluble CEA (data not shown) in competitive inhibition binding studies. However, at physiological concentrations in the long term (>24 h) *in vitro* assays in which equilibrium can be established, functional activity of the MEDI-565 is not impaired. We speculate that the assays showing tumour killing are more complex than receptor–ligand binding interactions (e.g., competitive inhibition binding). Nevertheless, the fact that a range of soluble CEA protein up to 1000 ng ml^−1^ could not block MEDI-565-induced T-cell cytotoxicity suggests that endogenous circulating CEA may not impair the clinical activity of MEDI-565. Furthermore, CEA-specificity studies were conducted by Lutterbuese *et al* (2009) showing that the MEDI-565 does indeed bind to CEA expressed on tumour.

Finally, because the molecular mechanism of T-cell cytotoxicity induced by BiTE molecules is still not fully determined, we performed additional studies and elucidated a role for granzyme B and perforin-mediated cell killing (Supplementary Figures 2 and 3). Gruen *et al* (2004) reported that bispecific antibodies against CD19 and CD3 (bscCD19xCD3) induced B-cell lysis in a perforin-dependent, but death receptor-independent manner. Offner *et al* (2006) analysed the immunological synapses of EpCAM-BiTE, and showed the localisation of perforin and granzyme in lytic synapses by immunofluorescence labelling. We also observed increased percentages of granzyme B-positive CD8/CD4 T cells beginning on day 2 and thereafter following the initiation of tumour/T-cell co-incubation in the presence of MEDI-565 (Supplementary Figure 2). Importantly, MEDI-565 significantly enhanced granzyme B secretion by T cells from the early time point of co-incubation, and gradually increased perforin levels in tumour/T-cell culture. Induction of apoptotic cell death in the AsPC-1 cell line corresponded well with this increase of perforin level, suggesting that MEDI-565 induces perforin/granzyme B-dependent T-cell cytotoxicity. Moreover, the addition of EGTA to the culture significantly inhibited MEDI-565-mediated T-cell cytotoxicity (data not shown), supporting the involvement of perforin, which requires extracellular calcium for pore formation (Lowin *et al*, 1996; Voskoboinik *et al*, 2005). However, one criticism of this proposed mechanism is that granzyme-mediated killing should be rapid. One unusual finding in our study was that the cell death associated with MEDI-565 occurred with a relatively slow onset (Figure 3), and the peak of the tumour cell death was detected on days 4 to 5 after the initiation of the tumour cell/T-cell co-incubation with MEDI-565. This is unlike other reports of bispecific antibodies of the BiTE class (Hoffmann *et al*, 2005; Hammond *et al*, 2007). On the basis of Supplementary Figure 2, it is possible that a critical concentration of perforin must be present to allow sufficiently released granzyme B to accumulate in target cells. We also found high-level secretion of several cytokines, including IFN-*γ* and TNF-*α* (Supplementary Figure 4), and further analyses are needed to elucidate the role of these cytokines in MEDI-565-redirected T-cell cytotoxicity.

In summary, human metastatic colorectal cancer, which had survived previous systemic chemotherapy, was sensitive to T-cell-mediated killing as shown using the novel CEA-specific BiTE MEDI-565. We observed apoptosis induction as well as anti-proliferative activity on tumour cells cultured with MEDI-565 and T cells, and showed that perforin/granzyme B had a key role in cytotoxicity. As CEA is expressed by many different kinds of cancers, MEDI-565 may be applicable to a wide range of adenocarcinoma as a novel treatment with the potential for high efficacy and less toxicity than conventional therapies. The next studies will be *in vivo* models to confirm activity against implanted colorectal cancers and if the results continue to show anti-tumour activity, these data would support the initiation of clinical trials with this therapy.
