# Supplementary material for: Metastatic colorectal cancer cells from patients previously treated with chemotherapy are sensitive to T-cell killing mediated by CEA/CD3-bispecific T-cell-engaging BiTE antibody
Source: Br J Cancer. 2009 Dec 1;102(1):124–33. doi: 10.1038/sj.bjc.6605364 (PMC2813763; doi:10.1038/sj.bjc.6605364)
Supplement: Supplementary Figures 1–4 [file 6605364x1.ppt]

## Slide 1
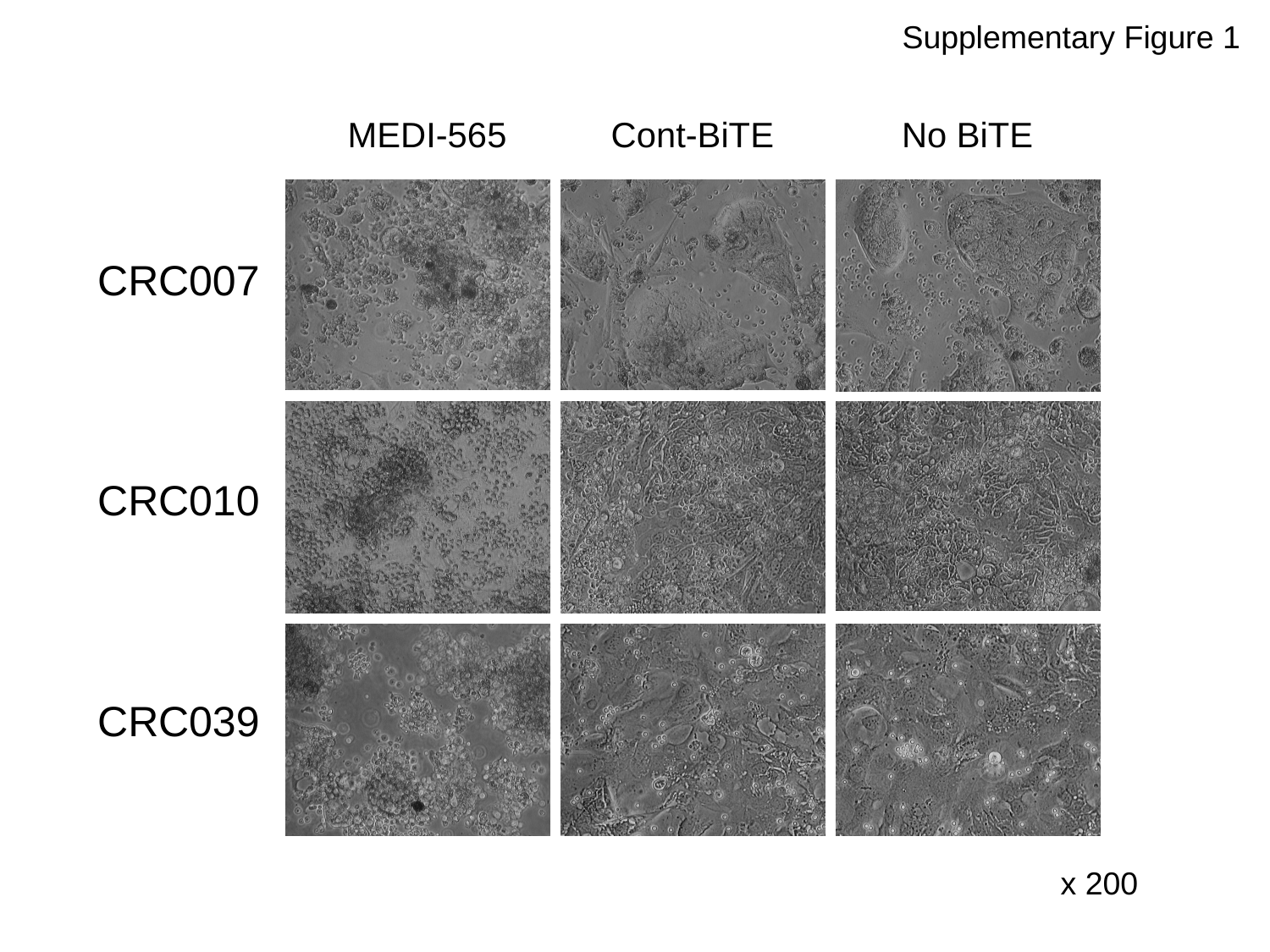

Supplementary Figure 1
MEDI-565
Cont-BiTE
No BiTE
CRC007
CRC010
CRC039
x 200

## Slide 2
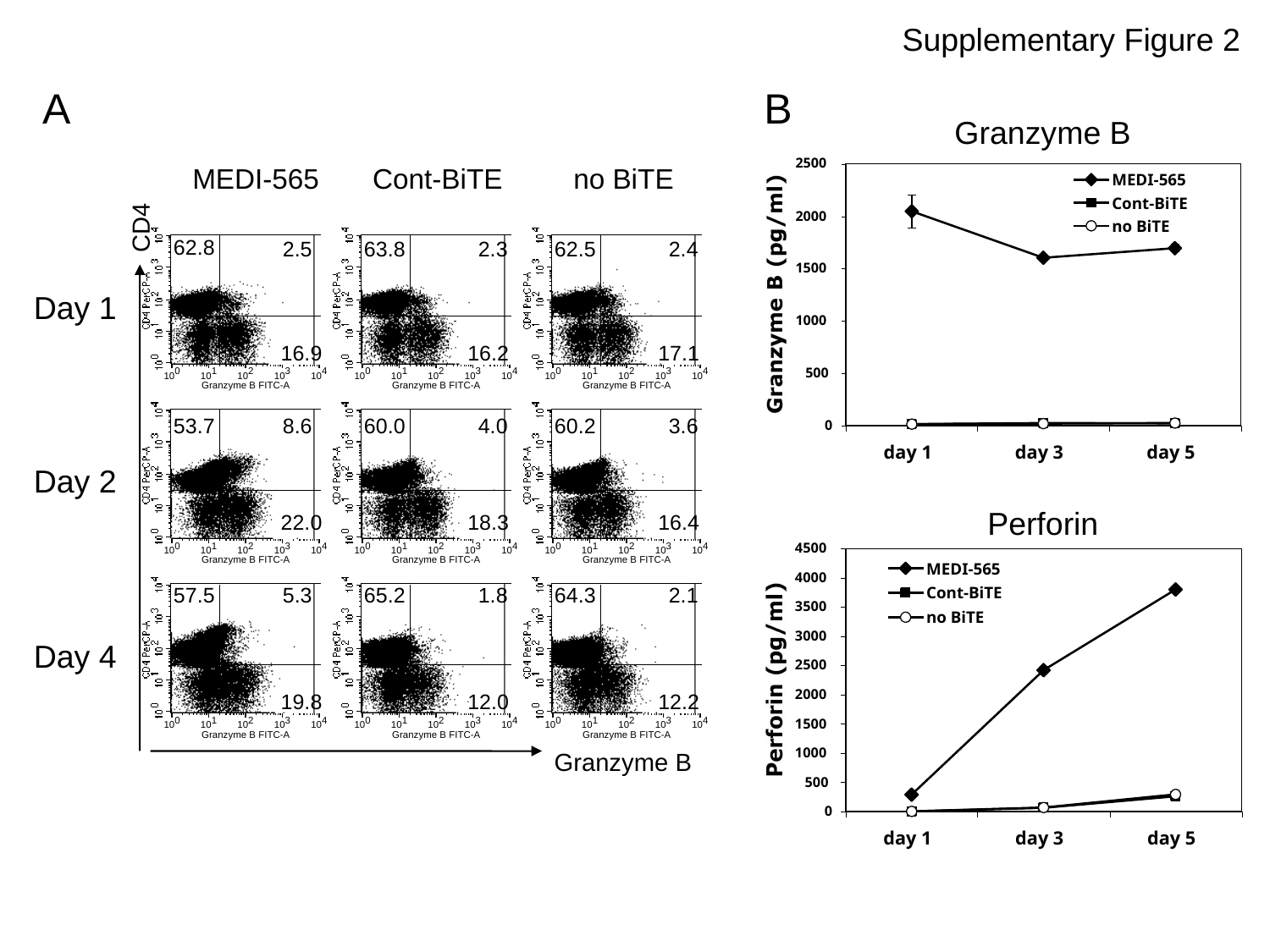

Supplementary Figure 2
A
B
Granzyme B
Perforin
MEDI-565
Cont-BiTE
no BiTE
CD4
62.8
2.5
63.8
2.3
62.5
2.4
Day 1
16.9
16.2
17.1
53.7
8.6
60.0
4.0
60.2
3.6
Day 2
22.0
18.3
16.4
57.5
5.3
65.2
1.8
64.3
2.1
Day 4
19.8
12.0
12.2
Granzyme B

## Slide 3
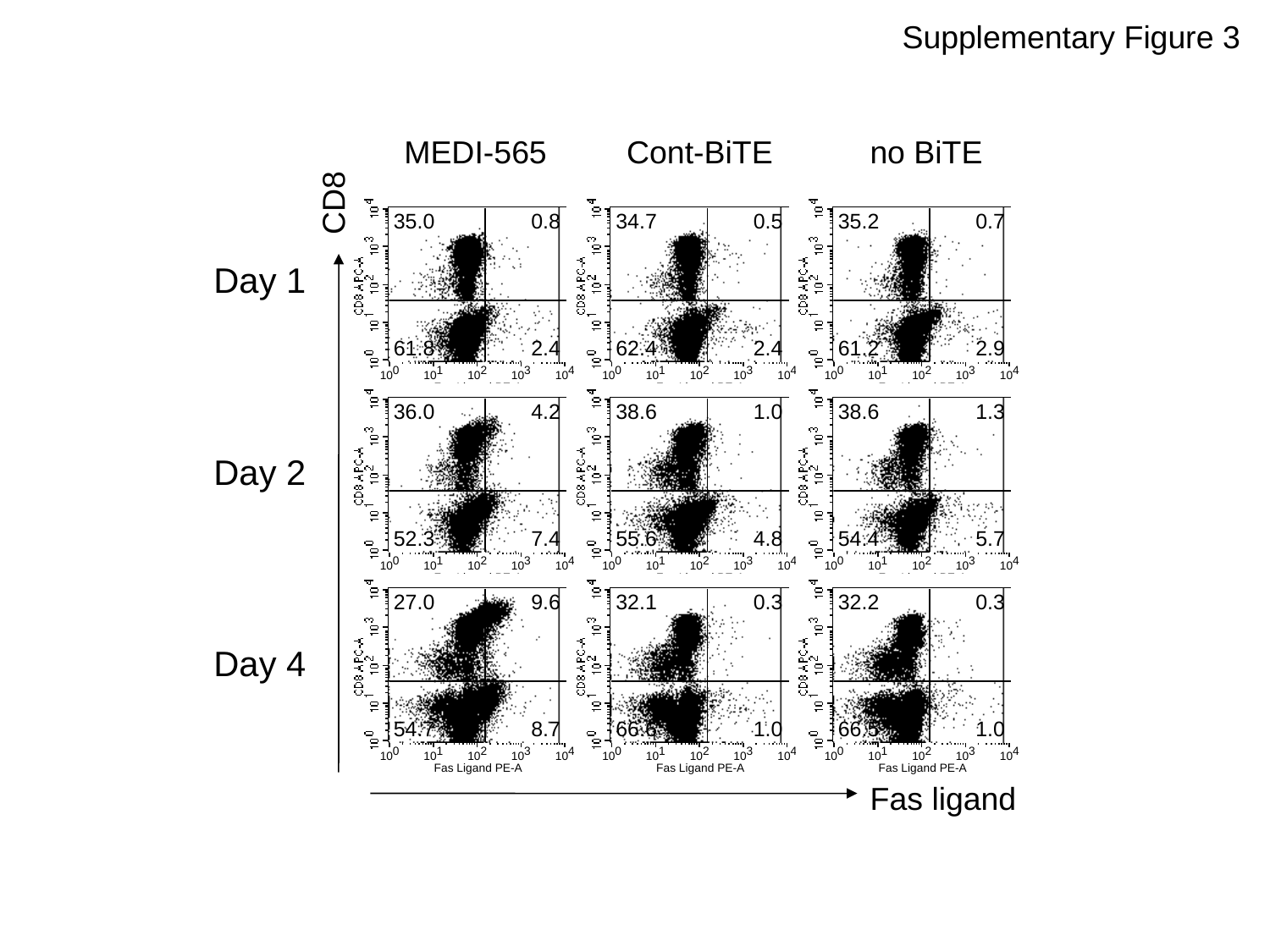

Supplementary Figure 3
Cont-BiTE
no BiTE
MEDI-565
CD8
35.0
0.8
61.8
2.4
34.7
0.5
62.4
2.4
35.2
0.7
61.2
2.9
36.0
4.2
52.3
7.4
38.6
1.0
55.6
4.8
38.6
1.3
54.4
5.7
27.0
9.6
54.7
8.7
32.1
0.3
66.6
1.0
32.2
0.3
66.5
1.0
Day 1
Day 2
Day 4
Fas ligand

## Slide 4
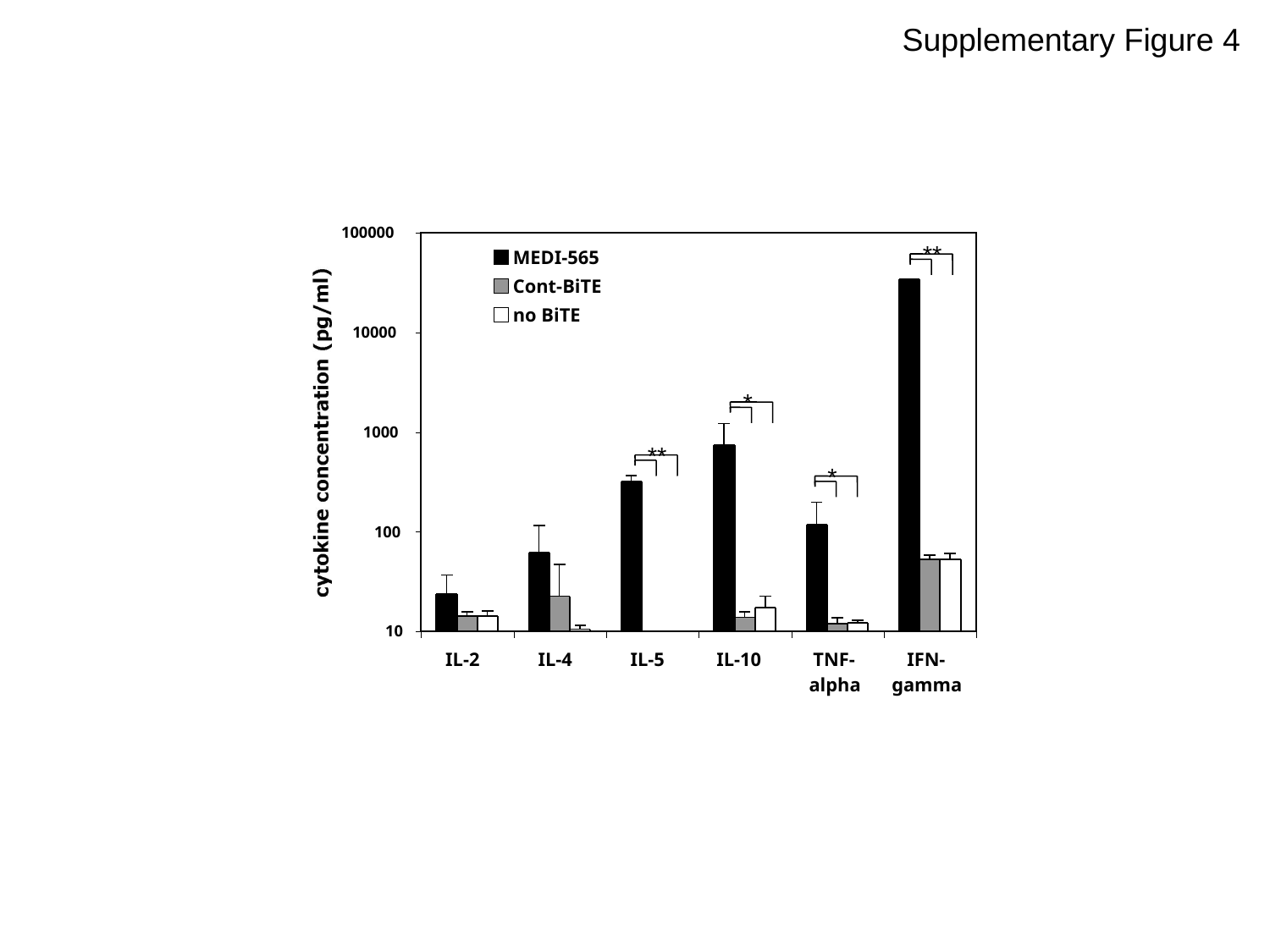

Supplementary Figure 4
**
*
**
*
