# Supplementary material for: Metastatic colorectal cancer cells from patients previously treated with chemotherapy are sensitive to T-cell killing mediated by CEA/CD3-bispecific T-cell-engaging BiTE antibody
Source: Br J Cancer. 2009 Dec 1;102(1):124–33. doi: 10.1038/sj.bjc.6605364 (PMC2813763; doi:10.1038/sj.bjc.6605364)
Supplement: Supplementary Figure Legends [file 6605364x2.doc]

**Supplementary Figure 1. Morphological changes of CEA+ human metastatic colorectal cancer explants induced by MEDI-565/T-Cell**

Colorectal cancer cells from liver metastatic lesions were surgically excised, implanted into NOD/SCID mice, and then maintained in in vitro culture as described in the Materials and Methods. Colorectal cancer cells were put into wells of 12 well plates (5 x 10^5^ cells/well) and allowed to adhere to the plate during overnight incubation. T cells were added to the well (2.5 x 10^6^ cells/well) together with MEDI-565 or MEC14 control BiTE (100 ng/mL). After 5 days incubation, photographs were taken.

**Supplementary Figure 2. MEDI-565 increased the Granzyme B-positive T cell population and Granzyme B/Perforin levels in the culture**

**A.** T cells were co-incubated with AsPC-1 cells with MEDI-565 or Cont BiTE (100 ng/mL) at a 5:1 effector-to-target (E:T) ratio for 1, 2 or 4 days. At the end of incubation time, Brefeldin A (1 mg/mL) was added to the culture. Cells were fixed, permeabilized with permeabilizing solution, and stained with anti-granzyme B-FITC, anti-CD4-PerCP, anti-CD8-APC. Lymphocytes were gated based on forward scatter/side scatter histograms and CD8+ or CD4+ cells were analyzed for granzyme B staining. Percentages of each quadrant are shown in each dot plot. **B.** T cells and AsPC-1 cells (E:T ratio=5:1) were co-incubated with MEDI-565 or Cont BiTE (100 ng/mL). Culture supernatants were harvested on days 1, 3 and 5. ELISA for granzyme B (upper panel) and perforin (lower panel) were performed.

**Supplementary Figure 3.** **MEDI-565 increased the Fas ligand-positive T cell population in the culture**

T cells were co-incubated with AsPC-1 cells with MEDI-565 or MEC14 control BiTE (100 ng/mL) as described in Figure 5 legend. Cells were fixed, permeabilized and stained with anti-Fas ligand-PE, anti-CD4-PerCP, and anti-CD8-APC antibodies. Lymphocytes were gated based on forward scatter/side scatter histograms and CD8+ and CD4+ cells were analyzed for Fas ligand staining. Percentages of each quadrant are shown in each dot plot.

**Supplementary Figure 4. MEDI-565 increased Tc1/Tc2 cytokine levels in the culture**

On day 5, culture supernatants were collected from wells containing tumor cells mixed with T cells in medium alone, or in medium supplemented with MEDI-565 or Cont BiTE, and tested for the levels of IL-2, IL-4, IL-5, IL-10, TNF-α and IFN-γ using a BD Cytometric Bead Array Th1/Th2 cytokine kit. **p<* 0.05, ***p<* 0.001 (Student’s *t* test).
